# Neuronal Changes in the Diabetic Cornea: Perspectives for Neuroprotection

**DOI:** 10.1155/2016/5140823

**Published:** 2016-12-01

**Authors:** Guzel Bikbova, Toshiyuki Oshitari, Takayuki Baba, Shuichi Yamamoto

**Affiliations:** Department of Ophthalmology and Visual Science, Chiba University Graduate School of Medicine, Inohana 1-8-1, Chuo-ku, Chiba, Chiba 260-8670, Japan

## Abstract

Diabetic neuropathy is associated with neurotrophic ulcerations of the skin and cornea. Decreased corneal sensitivity and impaired innervation lead to weakened epithelial wound healing predisposing patients to ocular complications such as corneal infections, stromal opacification, and surface irregularity. This review presents recent findings on impaired corneal innervation in diabetic individuals, and the findings suggest that corneal neuropathy might be an early indicator of diabetic neuropathy. Additionally, the recent findings for neuroprotective and regenerative therapy for diabetic keratopathy are presented.

## 1. Introduction

Diabetes mellitus is a major disease worldwide, and the incidence of diabetes has risen markedly in the past several decades. The complications associated with diabetes are the leading cause of blindness in the working age adults. Diabetic neuropathies characterized by a progressive loss of nerve fibers are common complications affecting about 50% of patients with diabetes [1].

It was recently demonstrated that retinal neuronal components were associated with the pathogenesis of diabetic retinopathy [2, 3], and the neuronal degeneration in the retina may be dependent on the mitochondrial- and caspase-dependent cell-death pathway [4]. It is known that neuronal abnormalities directly affect visual function in diabetic retinopathy, and those neuronal changes are probably also the reason for diabetic keratopathy, as cornea is one of the most highly innervated tissues [5]. However, there are limited numbers of studies available that focused on evaluation of corneal innervation changes in diabetic patients. Early diagnosis of neuropathy is very important for evaluation of risks and therapeutic management. Thus in this review we aim to summarize the most recent findings on impaired corneal innervation in diabetic patients and findings on neuroprotective and regenerative therapy approach for diabetic keratopathy.

## 2. Corneal Innervation

The density of corneal epithelial nerves is 300–600 times higher than that of the skin with approximately 7000 nociceptors/mm^2^ [6]. The sensory nerve fibers in the peripheral cornea have a myelinated shell and can be seen by slit-lamp biomicroscopy. The central cornea is acutely sensitive especially along the horizontal meridian and less sensitive along the vertical meridian [7].

The ophthalmic nerve and occasionally the maxillary branch of the trigeminal nerve innervate the cornea [8, 9], and the superior cervical ganglion supplies sympathetic innervation to the limbus and peripheral cornea [10]. Light [10–12] and electron microscopic [13, 14] studies and more recently confocal microscopic studies [15–18] have provided detailed information on the distribution of the nerves in the human cornea.

There are about 70 to 80 large diameter myelinated nerves that enter the cornea at the posterior to mid-stromal level, and they run radially and anteriorly toward the center of the cornea. The anterior stromal layers are innervated by multiple branches of these nerves that have no perineurium and myelin. They penetrate the cornea approximately 1 mm from the limbus, pass through Bowman's membrane, and turn in a clockwise direction forming the subbasal nerve plexus that lies between Bowman's layer and the epithelium forming the subbasal nerve vortex. Its geographic center is located between 2.18 and 2.92 mm from the corneal apex. In some cases, the subbasal nerves do not form a prominent spiral but end on opposite sides of an imaginary interface [13].

The functioning of the corneal nerve is assessed by corneal sensitivity tests. There are three main groups of receptors in the cornea: mechanical or mechanonociceptors, chemical or polymodal nociceptors, and thermal or cold receptors [19, 20].

## 3. Mechanisms of Impaired Corneal Innervation and Neurotrophic Role of Corneal Nerves

Recent studies have provided strong evidence that glycation plays an important role in the pathogenesis of retinal diabetic neuropathy with the triggering of different mechanisms that result in neuronal dysfunction [21, 22]. Obrosova and Julius reported that oxidative stress and poly(ADP-ribose) polymerase activation were fundamental mechanisms that play a role in the pathogenesis of diabetic neuropathy [23]. Byun et al. confirmed that poly(ADP-ribose) polymerase inhibition prevented the loss of epithelial innervation and promoted epithelial wound healing. They concluded that poly(ADP-ribose) polymerase activation played a role in the pathogenesis of diabetic neuropathy [24].

The results of a recent study have shown that one of the functions of corneal nerve fibers is in maintaining a healthy cornea and promoting wound healing after eye injuries [5]. The results of* in vitro* studies suggested that there is a trophic support between corneal epithelial cells and neurons. For example, trigeminal neurons release neurotransmitters and neuropeptides to provide for corneal epithelial cell growth, proliferation, differentiation, and type VII collagen production [25, 26]. Accordingly, corneal epithelial cells release soluble factors such as NGF and GDNF that stimulate neurite survival and extension [27–29]. Lambiase et al. reported that stromal keratocytes also produce neurotrophins, for example, neurotrophins 3 and 4 [30–32]; however their trophic influences on corneal nerve fibers still remain undetermined. BDNF is found in the corneal epithelial cells and stromal keratocytes, and it is believed to be also contained in corneal sensory neurons [24, 32].

Nerve-derived trophic factors regulate the biochemistry of the corneal epithelium and control the normal and renewal processes of maintaining the corneal epithelial cells. Thus, patients with impaired corneal innervation, for example, after herpetic keratitis, diabetes, prolonged contact lens wear, advanced age, and refractive surgery, are at increased risk of corneal damage because of diminished trophic support [24, 33]. Ferrari et al. reported that nerve-secreted neuropeptides influence corneal cell proliferation* in vitro,* and the rate of corneal epithelial cell mitosis is altered in denervated corneas of rats [34]. Ciliary neurotrophic factor (CNTF) has been detected in corneal endothelial cells [35]. Zhou et al. discovered that the mRNA of CNTF was more significantly downregulated in diabetic mice than in normal mice [36]. A subconjunctival injection of CNTF significantly reduced the size of the corneal epithelial defect of 67.89 ± 12.27% to 30.10% ± 10.13% after 48 hours. These results suggest that impaired corneal epithelial wound healing in diabetic mice can be caused by reduced levels of CNTF, and CNTF supplementation can promote corneal epithelial wound healing by activating corneal epithelial stem/progenitor cells [36].

In a very recent study, Gao et al. 2016 demonstrated that dendritic cells mediate sensory nerve innervation and regeneration through CNTF, and diabetes reduces the dendritic cells populations in both normal and injured corneas. This then results in decreased CNTF and impaired sensory nerve innervation and regeneration [37]. They also suggested that diabetes disrupted the neural communications of dendritic cells resulting in diabetic neuropathy and impairs sensory nerve regeneration in the cornea. Thus, dendritic cell-based therapy should be explored for diabetic neuropathy [37].

A study of the effects of neuropathy showed that the diabetes-induced denervation of the cornea reduces the viability of the corneal epithelial cells and their ability to recover from injury [38]. Guo et al. reported that the trigeminal ganglion neurons and the innervation of the cornea were impaired in diabetic mice [39].

A detailed listing of the neurotrophic factors present in the cornea is shown in Table 1.

## 4. Corneal Sensitivity in Diabetes

Patients with diabetes have decreased corneal sensitivity and thus are very vulnerable to trauma. In the study by Nielsen it was demonstrated that corneal sensitivity (determined using Cochet and Bonnet's aesthesiometer) in 83% of diabetic patients was reduced below 60 mm against 38% of the controls alongside with reduced perception of vibrations (vibratory perception of the left index finger and great toe by biothesiometer) [55]. A decrease in corneal sensitivity may cause a delay in epithelial wound healing and be the cause of recurrent erosions. This is because the corneal nerves release epitheliotropic substances that promote the maintenance of the integrity of corneal surface [56]. Alterations of the corneal nerves decrease the corneal sensitivity resulting in corneal hypoesthesia that disrupts the epithelial architecture and function. These changes would further delay the reepithelization of the cornea.

Confocal microscopy has shown promise as a noninvasive method for quantifying the damage and repair of corneal sensory nerves that can serve as markers for diabetic neuropathy. Thus, Rosenberg et al. using confocal microscopy found decreased corneal sensitivity together with a decreased number of long nerve fiber bundles in the subbasal nerve plexus. In addition, patients with diabetes had fewer nerve fiber bundles than healthy control subjects possibly due to the presence of polyneuropathy. In all patients with diabetes with neuropathy, the subbasal nerve densities were significantly reduced [57]. Additionally, the authors found that even if most patients with diabetes had nerve fiber bundles with a normal morphology, patients under dialysis with mild to moderate neuropathy had abnormally tortuous nerve fiber bundles in the subbasal nerve plexus. This confirmed the presence of an impairment of corneal sensitivity, and the duration of diabetes was significantly and directly correlated with the degree of polyneuropathy.

In the subbasal nerve plexus, a decrease in the nerve density, number of branches, single nerve fiber length, and increased tortuosity have been found to be significantly correlated with established electromyography and nerve conduction parameters and with the results of the skin biopsy [58, 59].

Tavakoli et al. suggested using confocal microscopy in longitudinal studies to assess progression of diabetic neuropathy [60]. In their recent study it was shown that patients with diabetic autonomic neuropathy had a progressive and significant reduction of corneal nerve fiber density, branch density, and length compared to healthy control subjects [61]

Misra et al. also described a clinical application of in vivo confocal microscopy in patients with diabetes mellitus type 1 [62]. They measured the corneal nerve parameters in type 1 diabetics and controls and confirmed a decrease in the subbasal nerve density and corneal sensitivity in diabetic patients. They also found a significant relationship between corneal neuropathy and systemic neuropathy. They concluded that corneal neuropathy might be an early indicator of diabetic neuropathy because it preceded other clinical and electrophysiology tests of neuropathy [62]. Also Pritchard et al. reported application of confocal microscopy in assessment of diabetic polyneuropathy [63] by measuring the corneal nerve fiber length, ability of confocal microscopy to predict the development of diabetic polyneuropathy with 63% sensitivity and 74% specificity, for a corneal nerve fiber length threshold cutoff of 14.1 mm/mm^2^ was demonstrated [63].

Several studies have confirmed that the cornea innervations were altered in animal models of diabetes. Davidson et al. reported a 50% loss of corneal nerve fibers after 12 weeks of high fat diet in low-dose streptozotocin-induced diabetic rats [64]. Yin et al. described corneal changes in streptozotocin-induced type 1 diabetic rats. A 50% decrease in tear secretion was found after eight weeks of diabetes induction in SD rats, corneal sensitivity was decreased, and the corneal nerves had fewer branches and were thinner and shorter by 75% [65].

Ueno et al. found a decreased density of the corneal subbasal nerve plexus and corneal epithelial branches in leptin receptor mutant mice which are an accepted animal model of type 2 diabetes. The corneal subbasal nerves were more tortuous in these mutant mice than in normal mice [66].

Wang et al. reported delayed corneal wound healing in the Akita diabetic mice, a model of chronic complications of type 1 DM [67]. In a longitudinal study of corneal nerve density in a rat model of type 1 diabetes, Chen et al. found that density of nerves was initially increased in the subbasal plexus after 8 and 16 weeks of diabetes. However, the density remained unchanged in the stromal layer [68]. An increase could be explained by increased nerve tortuosity or collateral sprouting as reported in patients with impaired glucose tolerance [69].

It is difficult to translate the results from the streptozotocin-induced animals to type 1 human diabetes and that from the db/db mouse to type 2 human diabetes due to recessive homozygous mutation in the leptin receptor (fa/fa) [70, 71]. Yorek et al., 2015, studied C57Bl/6J mice fed a high fat diet that caused an elevated level of glucose in the fasting blood, and they demonstrated that the diet-induced obesity led to the development and progression of peripheral neuropathy and nerve structural damage in the cornea with or without hyperglycemia [72].

Diabetic neurotrophic keratopathy, a manifestation of diabetic polyneuropathy, also plays a significant role in limiting epithelial wound healing. Neurotrophic keratopathy according to Mackie [73] has three stages: Stage 1, positive Bengal staining of the inferior palpebral conjunctival surface, punctate keratopathy, and a decrease in tear breakup time; Stage 2, epithelial breakdown with epithelial deficits surrounded by loose epithelium that becomes hazy, edematous, and poorly adherent to Bowman's layer; and Stage 3, corneal ulceration that can lead to melting and perforation of the cornea.

## 5. Therapeutical Management

A diabetic corneal ulcer is a challenging clinical condition. The development of persistent corneal epithelial defects is often associated with severe peripheral neuropathy. The success in the management of neurotrophic keratopathy is generally based on achieving epithelial healing and preventing a progression of the corneal damage. Pathogenetic-orientated pharmacological treatment for neurotrophic keratopathy is still not available. Preservative-free artificial tears, topical antibiotics, bandage contact lenses, amniotic membrane transplantation, tarsorrhaphy, and a conjunctival flap are useful methods that have been used to treat refractory neurotrophic corneal ulcers [74]. Therefore, the establishment of a pathogenetic treatment that can lead to neuroprotection and neuroregeneration is extremely important.

Different nerve-secreted factors, such as NGF and BDNF, are important factors necessary for epithelial regeneration [75–77]. The application of exogenous NGF was able to reverse the damage of peripheral nerves and completely heal corneal epithelial defects in patients with neurotrophic keratitis [44]. According to Ueno, corneal stem/progenitor cells are closely associated with corneal wound healing and insulin-like growth factor-I administration may be a promising agent that can be used to prevent persistent corneal ulcers in patients with type 2 diabetes. Tavakoli et al. examined patients with type 1 diabetes by confocal microscopy after simultaneous pancreas-kidney transplantation, and they obtained evidence of an early regeneration of corneal nerves [78]. Their following study showed that continuous subcutaneous insulin infusion showed an improvement in corneal nerve morphology, consistent with small fiber regeneration (assessed by confocal microscopy), most probably due to more stable blood glucose control [79].

By accelerating neuronal cell death, diabetes can lead to inhibiting neurite regeneration. Dai et al., 2015, reported that the neurites were significantly longer in the neuropeptide FF-treated diabetic neurons compared with the nontreated controls in primary cultured diabetic trigeminal sensory neurons [80]. They concluded that neuropeptide FF provided nerve growth-promoting effects to the* db/db* mice through the ERK1/2 pathway which is known to play a central role in controlling Schwann cell plasticity and peripheral nerve regeneration [81].

Recent laboratory investigations have shown that new corneal nerve modulators, for example, pigment epithelial-derived factor (PEDF), with DHA enhance the regeneration of corneal nerves and the recovery of corneal sensitivity following corneal nerve damage [82].

Ishibashi et al. reported that a PEDF-derived synthetic modified peptide inhibits tubular cell damage through its antioxidative properties under diabetes-like conditions. This suggested that supplementation of modified P5-3 peptide may be a therapeutic strategy for diabetic nephropathy [83]. Oswald et al., 2012, suggested intranasal delivery of nanomicelle curcumin solution promoted corneal epithelial/nerve healing in diabetic mice. This was because the pharmaceutically active ingredient was delivered to the trigeminal ganglion neuron and thus helps in the diabetic corneal epithelial/nerve wound healing [38].

There are other reports that miRNAs, including miR-21 and miR-29b, can be used as therapeutic agents to stimulate peripheral nerve regeneration [84, 85]. Wang et al. reported that miR-182 can protect trigeminal neurons from peripheral nerve damage in an experimental mouse model of diabetes. They suggested that this was accomplished by its ability to enhance neurite outgrowth in isolated trigeminal sensory neurons. This overcame the detrimental effects of hyperglycemia by stimulating corneal nerve regeneration by decreasing the expression of one of its target genes, NOX4 [86].

Other approaches include gene therapy [87] and use of *α*-Lipoic Acid in Soluplus readily renders nanomicelles as alternative ocular treatment of diabetes-associated corneal diseases had been reported recently [88].

## 6. Conclusions

Impaired corneal innervation in diabetes is an important early indicator of diabetic neuropathy. The decrease in corneal sensitivity with a longer duration of diabetes is correlated with the severity of neuropathy. Further investigations on mechanisms of corneal nerve damage and establishment of pathogenetic-orientated therapy for diabetic neuropathy should be the main focus of future research.

## Figures and Tables

**Table 1:** Neurotrophic factors in the cornea.

Growth factor	Healthy cornea	Injured cornea	Topical application
Nerve growth factor (NGF)	(i) Found in corneal epithelium and stromal keratocytes (ii) Critical for corneal nerve survival and maintenance, axonal branching, elongation, neuronal sprouting, and regeneration following nerve damage [40]	Upregulated during reinnervation after nerve surgical transection [41], and in dry eye syndrome [42], in inflamed conjunctiva of patients with vernal keratoconjunctivitis [43]	(i) Augments corneal wound healing and provides recovery of corneal sensitivity and photophobia [44] (ii) Has potent antiviral properties (restrict herpes simplex virus-1 [45])

Brain-derived neurotrophic factor (BDNF)	(i) Found in corneal epithelium and stromal keratocytes, originate from corneal sensory neurons [32] (ii) Exact role related to corneal nerves is unclear	Expressed after experimental flap surgery in putative corneal stromal and/or inflammatory cells in a positive association with neurite extension [40]	

Glial cell-derived neurotrophic factor (GDNF)	Expressed in human corneal stromal keratocytes and may operate similarly to or synergistically with NGF by triggering gene transcription governing epithelial cell migration and wound healing [32]	Possibly plays an important role in corneal regeneration and wound healing [46]	Produces complete epithelial healing in a patient with a progressive neurotrophic ulcer [46]

Neurotrophins 3, 4/5 (NT-3, NT-4/5)	(i) NT-3 transcribed in epithelial cells and stromal keratocytes [32] (ii) NT-4 is present in corneal epithelium and is a neurotrophic factor that may be involved in the regulation of stromal keratocytes by epithelial cells [32]	Minimal changes in NT-3 gene expression following surgical transection of corneal nerves [41]	

Ciliary neurotrophic factor (CNTF)	Promotes corneal epithelial wound healing by activating corneal epithelial stem/progenitor cells [47]	(i) Upregulated in corneal epithelium after injury in mice [47] (ii) Downregulated in corneal epithelium of diabetic mice [36]	

Vascular endothelial growth factor (VEGF)	Minimally present [48]	(i) Upregulated in the injured cornea [48] (ii) Required for efficient corneal nerve regeneration	VEGF supplementation promotes trigeminal nerve repair, and abrogation of VEGF signaling reduces corneal nerve growth [48, 49]

Hepatocyte growth factor (HGF)	Expressed in stromal keratocytes, stimulates corneal epithelial proliferation [50]	Upregulated after corneal epithelial wounding and probably contributes to the epithelial wound healing process [49]	

Keratocyte growth factor (KGF)	(i) Expressed in stromal keratocytes [32], fibroblasts [51] (ii) Stimulates corneal epithelial proliferation, acts specifically on cells of epithelial origin as a paracrine mediator [51]	Upregulated in corneal epithelial wounding [52]	

Transforming growth factor-*α* (TGF-*α*), interleukin-1*β* (IL-1*β*), and platelet-derived growth factor-B (PDGF-B)	(i) Exclusively expressed in the corneal stroma [53] (ii) TGF-*α* and IL-1*β* can upregulate the transcription of neurotrophins, such as NGF in 3T3 mouse fibroblasts [54]		
